# Treatment of poor placentation and the prevention of associated adverse outcomes – what does the future hold?

**DOI:** 10.1002/pd.4401

**Published:** 2014-05-29

**Authors:** RN Spencer, DJ Carr, AL David

**Affiliations:** Institute for Women's Health, University College LondonLondon, UK

## Abstract

**ABSTRACT:**

Poor placentation, which manifests as pre-eclampsia and fetal growth restriction, is a major pregnancy complication. The underlying cause is a deficiency in normal trophoblast invasion of the spiral arteries, associated with placental inflammation, oxidative stress, and an antiangiogenic state. Peripartum therapies, such as prenatal maternal corticosteroids and magnesium sulphate, can prevent some of the adverse neonatal outcomes, but there is currently no treatment for poor placentation itself. Instead, management relies on identifying the consequences of poor placentation in the mother and fetus, with iatrogenic preterm delivery to minimise mortality and morbidity. Several promising therapies are currently under development to treat poor placentation, to improve fetal growth, and to prevent adverse neonatal outcomes. Interventions such as maternal nitric oxide donors, sildenafil citrate, vascular endothelial growth factor gene therapy, hydrogen sulphide donors, and statins address the underlying pathology, while maternal melatonin administration may provide fetal neuroprotection. In the future, these may provide a range of synergistic therapies for pre-eclampsia and fetal growth restriction, depending on the severity and gestation of onset.

## INTRODUCTION

In normal pregnancy, trophoblast invasion of the maternal spiral arteries produces a low-resistance, high-flow maternal uterine circulation. These changes are enhanced by the placental production of vasoactive substances, such as vascular endothelial growth factor (VEGF) and placental growth factor (PlGF). VEGF and, to a lesser extent, PlGF cause angiogenesis and activate endothelial nitric oxide synthase (eNOS) to produce nitric oxide (NO) and hence vasodilatation. In contrast, poor placentation appears to arise from interactions between inadequate trophoblast invasion, leading to reduced placental perfusion, and oxidative stress, resulting in the production of inflammatory cytokines.^1^,^2^ Recent evidence from a study of placental bed biopsies in pregnancies affected by fetal growth restriction (FGR) and pre-eclampsia confirms that there is a major defect in myometrial spiral artery remodelling that is linked to clinical parameters.^3^ Although the sequence and relative contributions of the defects are unclear, the result is a relative reduction in uterine artery blood flow,^3^,^4^ an increase in the soluble VEGF receptor soluble fms-like tyrosine kinase 1 (sFlt-1), and a reduction in the available maternal VEGF and PlGF.^1^,^2^,^5^,^6^ Other synergistic antiangiogenic proteins such as soluble endoglin are increased, leading to inhibition of transforming growth factor-β signalling. The phenotype is thus attributable to an antiangiogenic state.^7^

Other articles in this review have considered whether we can predict and prevent poor placentation. This article will focus on strategies to treat poor placentation and prevent the adverse outcomes it produces.

## HOW CAN WE TREAT POOR PLACENTATION?

**Figure 1:**
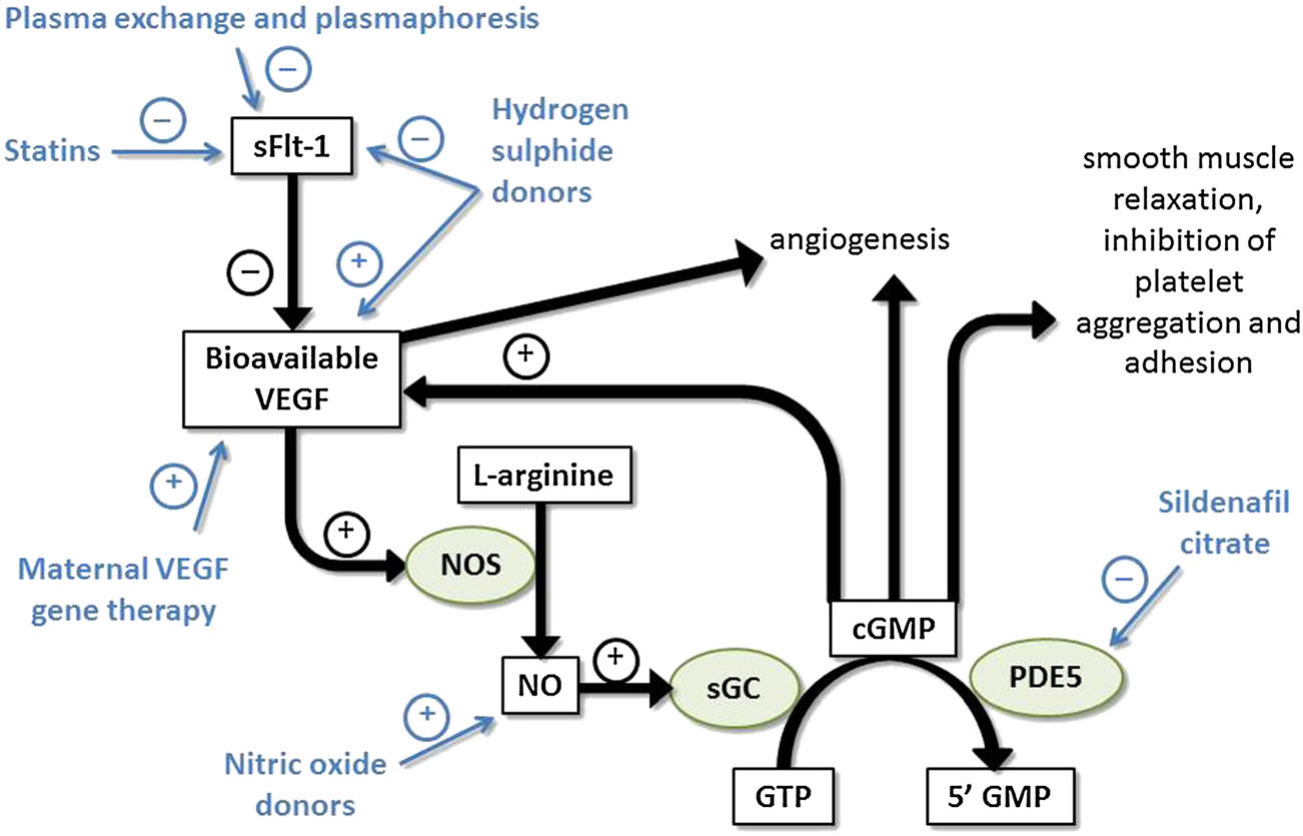
Sites of action of the interventions currently under investigation as treatments for established poor placentation. sFlt-1, soluble fms-like tyrosine kinase 1; VEGF, vascular endothelial growth factor; NOS, nitric oxide synthase; NO, nitric oxide; sGC, soluble guanylate cyclase; GTP, guanosine-5′-triphosphate; cGMP, cyclic guanosine monophosphate; 5′ GMP, guanosine monophosphate; PDE5, phosphodiesterase type 5 inhibitor

Currently, there is no treatment for poor placentation once it has developed. The maternal manifestations of pre-eclampsia may be manageable in the short term, but the only way to cure pre-eclampsia, and prevent stillbirth and severe *in utero* neurological damage in FGR, is to deliver the fetus and placenta. Depending upon gestation, this may mean very preterm delivery, with its own associated complications. Numerous interventions have been investigated, including bed rest, betamimetics, calcium channel blockers, oxygen or nutrient supplementation, and antioxidants such as vitamins C and E. In all cases, systematic reviews have not found sufficient evidence to evaluate or recommend their use, possibly because they do not address the root of the problem: antiangiogenesis.^8^–^14^ Despite the many challenges in developing obstetric therapies and relative underinvestment from the pharmaceutical industry, a number of promising interventions are now emerging, many from academic-industrial partnerships (Figure 1).

### Nitric oxide donors

Several studies have looked at the effects of the organic nitrates glyceryl trinitrate and isosorbide dinitrate in women with pre-eclampsia.^15^ These have demonstrated a reduction in maternal blood pressure, uterine artery pulsatility index and resistance index, and umbilical artery resistance index with no evidence of adverse effects on fetal heart rate. The studies have been small, however, with only three randomised trials, which were powered to look at short-term haemodynamic changes rather than outcome.^16^–^18^ Furthermore, organic nitrates are associated with tolerance, requiring nitrate-free periods. An alternative NO donor, S-nitrosoglutathione (GSNO), does not produce tolerance, reduces platelet aggregation, and increases the antioxidant glutathione.^15^ A phase I study in women with early-onset pre-eclampsia (21–33 weeks of gestation, *n* = 10), in which eight of the fetuses were classified as growth restricted, found that GSNO significantly reduced maternal blood pressure and uterine artery resistance index without affecting fetal Doppler measurements.^19^ Furthermore, a recent dose ranging study found that GSNO could reduce small vessel tone, platelet activation, and proteinuria at doses that did not significantly reduce maternal mean arterial pressure.^20^ Larger studies are now needed to determine whether NO donors can produce clinically meaningful differences in outcome.

### Sildenafil citrate

The phosphodiesterase-5 inhibitor sildenafil produces its vasodilatatory effects by preventing the breakdown of NO. Initial evidence to support its use as a therapy for poor placentation came from *in vitro* myography studies; myometrial vessels from women with pre-eclampsia exhibited significantly less relaxation than healthy controls, but relaxation increased after exposure to sildenafil citrate.^21^ These findings were replicated in myometrial vessels from pregnancies affected by FGR in the absence of pre-eclampsia.^22^ Unfortunately, results from a double-blind randomised placebo-controlled trial in women diagnosed with early-onset pre-eclampsia (24–34 weeks of gestation, *n* = 35) were disappointing.^23^ Sildenafil was well tolerated, with no increase in maternal or fetal adverse events, but it did not increase the interval to delivery, possibly because the pathological process was too advanced. In addition, a dose escalation design meant over half of the women received only the lowest dose of 20 mg three times a day (TDS). No difference was found in the vascular reactivity of myometrial vessels from women receiving sildenafil compared with placebo, possibly because of the interval between the last dose and delivery.^24^

Results of more recent pre-clinical studies, however, have been promising. The catechol-O-methyltransferase (COMT-/-) knockout mouse model of poor placentation results in increased umbilical artery pulsatility index (UAPI) and smaller and lighter pups compared with controlled mice.^25^ However, COMT-/- mice given sildenafil from 12.5 to 18.5 days postconception (dpc) had significantly higher pup weight, crown-rump length, and abdominal circumference (AC) than untreated COMT-/- mice and significantly lower UAPI. This effect of sildenafil on fetal growth was not replicated in a study using the rat reduction of uteroplacental perfusion pressure (RUPP) model, which involves clipping the maternal aorta and ovarian arteries at 14 dpc (term = 22 dpc), resulting in maternal hypertension, an increase in sFlt-1 and oxidative stress, and a reduction in free VEGF and fetal weight.^26^ However, RUPP dams receiving sildenafil did have a significantly lower mean arterial pressure than untreated RUPP dams.

The potential for sildenafil to improve fetal growth was investigated in an open-label pilot study.^27^ Ten women with pregnancies affected by severe early-onset ‘dismal prognosis’ FGR, where the chance of intact fetal survival was felt to be less than 50%, accepted the option of taking 25-mg sildenafil TDS. Outcomes were compared with those from matched contemporaneous sildenafil-naive pregnancies (*n* = 17). Sildenafil treatment was associated with increased posteligibility fetal growth velocity in the AC [9/10 (treated) vs 7/17 (naive); odds ratio, 12.9; 95% CI, 1.3, 126]. In the tenth sildenafil-treated pregnancy, where umbilical artery end diastolic flow was already reversed, stillbirth ensued within 24 h of starting the intervention. No maternal adverse effects were reported, although a greater proportion of treated women developed pre-eclampsia. Overall, there was a trend towards improved perinatal survival in the sildenafil-treated group; however, it is unclear if the higher levels of termination and permissive stillbirth in the sildenafil-naive group reflect poorer prognosis or altered management.

The effects of sildenafil in early-onset FGR are being addressed by the STRIDER NZAus trial, starting shortly in Australia and New Zealand (Table 1). This phase II double-blind randomised placebo-controlled trial will look at the effect of 50-mg oral sildenafil TDS on fetal growth velocity in FGR diagnosed before 30 weeks of gestation. Secondary outcomes will include rates of perinatal mortality and intact survival. A UK version of the trial is also in development.

**Table 1:** A summary of the clinical trials discussed in this article, which are currently investigating treatments for poor placentation and its associated adverse outcomes or are because of start

Trial registration	Trial name	Target population	Intervention	Trial design	Primary outcome	Status
ISRCTN23410175^a^	StAmP: statins to ameliorate early onset pre-eclampsia	Women with pre-eclampsia between 24 + 0 and 31 + 6 weeks of gestation	Oral pravastatin, 40 mg daily	Double-blind randomised placebo-controlled trial	Soluble fms-like tyrosine kinase 1 (sFlt-1) at 48 h postrandomisation	Started June 2011
NCT01717586^b^	Pravastatin for prevention of pre-eclampsia	Women 12 + 0 to 16 + 6 weeks of gestation with severe pre-eclampsia in either of the two preceding pregnancies	Oral pravastatin at 10 mg daily or 20 mg daily	Double-blind randomised placebo-controlled trial	Maternal, fetal and neonatal adverse events and pharmacokinetics	Started August 2012
ACTRN12612000584831^c^	STRIDER (NZAus): a randomised placebo-controlled trial of sildenafil therapy to improve fetal growth velocity in dismal prognosis early-onset intrauterine growth restriction (New Zealand and Australia)	Women with intrauterine growth restriction <30 + 0 weeks of gestation	Oral sildenafil, 50 mg three times a day	Phase II blinded randomised placebo-controlled trial	The proportion of pregnancies with increased abdominal circumference growth velocity	Not started
ACTRN12612000858897^c^	A pilot study of maternally administered melatonin to decrease the level of oxidative stress in human pregnancies affected by intrauterine growth restriction	Women with intrauterine growth restriction between 23 + 0 and 34 + 0 weeks of gestation	Oral melatonin, 4 mg twice a day	Single-arm, unmasked pilot study	Umbilical artery malondialdehyde and 8-isoprostane as markers of oxidative stress	Started September 2012
ACTRN12613000476730^c^	PAMPR: a pilot study of prenatal maternally administered melatonin to decrease the level of oxidative stress in human pregnancies affected by pre-eclampsia	Women with pre-eclampsia between 24 + 0 and 35 + 6 weeks of gestation	Oral melatonin, 10 mg three times a day	Single-arm, unmasked phase I trial	Interval from diagnosis to delivery	Not started
NCT01404910^b^	RAAPID-II: removal of antiangiogenic proteins in pre-eclampsia before delivery	Women with pre-eclampsia between 23 + 0 and 32 + 0 weeks of gestation	Aphaeresis using the Liposorber LA-15 System	Single-arm, unmasked phase 1b trial	Maternal sFlt-1 levels before and at given intervals after aphaeresis therapy	Started June 2013

Full details of the eligibility criteria and interventions can be found at the trial registries.

a www.clinicaltrialsregister.eu.

b www.clinicaltrials.gov.

c www.anzctr.org.au.

### Maternal VEGF gene therapy

An alternative approach to treating poor placentation is to increase the levels of VEGF in the maternal uterine arteries, thus improving local vasodilatation and angiogenesis. This can be achieved with an adenoviral (Ad) gene therapy vector, either injected into the uterine arteries or applied to the outside of the vessels, which produces short-term VEGF expression (Ad.VEGF). Studies in large and small animals have confirmed the efficacy of this approach for improving fetal growth in FGR. In normal sheep pregnancy, injection of Ad.VEGF (1 × 10^11^ particles), compared with injection of a control non-vasoactive vector (Ad.LacZ), increased uterine artery volume blood flow within 7 days of injection. VEGF expression in the perivascular adventitia of the treated vessels was associated with reduced uterine artery contractile response, increased uterine artery relaxation response, and increased expression of eNOS, inducible NOS, and VEGF receptor 2.^28^ In long term, this increase in flow persisted for at least 4 weeks until the end of gestation. Although no VEGF expression remained, the reduced contractile responses of the uterine artery persisted and periadventitial blood vessels were significantly increased, suggesting that the on-going improvement in uterine blood flow was mediated by vascular remodelling.^29^ Importantly, there was no evidence of vector spread or expression in fetal tissues and no effect of the vector on maternal or fetal haemodynamic measures. In a sheep model of FGR, overnourishment of pregnant adolescent ewes results in approximately half of the fetuses demonstrating marked asymmetric growth restriction with increased umbilical artery Doppler indices and reduced placental expression of VEGF.^30^–^32^ Overnourished adolescent dams were randomised to receive Ad.VEGF, Ad.LacZ, or saline, injected into both uterine arteries. The fetal growth velocity, as determined by ultrasound measurement of AC, was significantly increased in the Ad.VEGF group compared with the Ad.LacZ or saline groups by 3 to 4 weeks after injection, and there appeared to be amelioration of fetal brain sparing.^33^ At necropsy, significantly fewer fetuses demonstrated marked FGR in the Ad.VEGF group than in the Ad.LacZ or saline groups. Increased fetal growth velocity was confirmed in a second experimental group, administering overnourished adolescent dams uterine artery injections of either Ad.VEGF or saline.^34^ Results from treatment in FGR guinea pig pregnancy suggest a similar improvement in fetal growth velocity.^35^

In the clinical context, vector delivery into the uterine arteries could be achieved through interventional radiology, which is supported by the Royal College of Obstetricians and Gynaecologists as a prophylactic measure before delivery in women at high risk of postpartum haemorrhage.^36^ While this is more invasive than administering oral medication, it has the potential advantage of targeting vasoactive changes to the maternal uteroplacental circulation. The EVERREST Project, which started in 2013, aims to carry out a phase I/IIa clinical trial to assess the safety and efficacy of maternal uterine artery Ad.VEGF gene therapy for severe early-onset FGR. The project, funded by the European Union, involves a multinational, multidisciplinary consortium, including experts in bioethics, fetal medicine, fetal therapy, obstetrics, and neonatology.

### Hydrogen sulphide donors

Hydrogen sulphide (H_2_S), like NO, is a vasorelaxant and pro-angiogenic gas. H_2_S produces vasodilatation by acting on smooth muscle cell adenosine triphosphate-sensitive potassium channels, while its angiogenic effects appear to be mediated by VEGF and the VEGF receptor 2.^37^ Placentas from pregnancies affected by FGR or pre-eclampsia with abnormal umbilical Dopplers have reduced expression of the enzyme cystathionine-gamma-lyase, which produces H_2_S.^38^ The H_2_S donor sodium hydrosulfide (NaHS) decreases vascular resistance in healthy placentas^38^ and mitigates the antiangiogenic effects of sFlt-1 *in vitro*.^39^ In rats with a pre-eclampsia-like phenotype induced by systemic Ad.sFlt-1 vector, intraperitoneal injection of NaHS was associated with significantly reduced maternal hypertension, proteinuria, and sFlt-1 levels and significantly increased VEGF levels compared with inert control injections.^39^ This was without any apparent adverse effects on pup growth or development. Further work is now needed to investigate the therapeutic potential of H_2_S donors in poor placentation.

### Statins

3-Hydroxyl-3-methylglutaryl coenzyme A reductase inhibitors, or statins, are best known for their lipid-modifying cardioprotective effects. However, they are also antiinflammatory, antioxidant, and may beneficially modify the pathways of poor placentation.

In mice administered Ad.sFlt-1 at 8 dpc, which produces a pre-eclampsia-like phenotype and increased vascular reactivity, maternal oral pravastatin from 9 to 18 dpc reduced the elevated sFlt-1 levels and reduced vascular contractility at 18 dpc (term = 21 dpc) compared with untreated Ad.sFlt-1 mouse controls.^40^ Similarly in the rat RUPP model, oral pravastatin administration to RUPP dams from 14 to 19 dpc ameliorated maternal hypertension and resulted in decreased sFlt-1, increased free VEGF, and increased fetal weight compared with untreated RUPP dams, giving comparable results to normal pregnant controls.^41^

Early concerns about possible teratogenicity with statins have not been supported by more recent population exposure studies, which have found no increase in congenital anomalies with statin use in pregnancy.^42^,^43^ Pravastatin is the most hydrophilic statin, which should limit placental transfer, but even so some transplacental passage does occur.^44^ Because statins increase NO bioavailability, this could potentially interfere with the preferential redistribution of fetal cardiac output to the brain, which is often seen with poor placentation. To address this question, the effect of intravenous fetal pravastatin administration was studied in sheep before, and 24 h after, 30 min of maternal acute hypoxia, designed to achieve a 50% reduction in the partial pressure of oxygen in the fetus.^45^ Although fetal exposure to pravastatin reduced indices of anaerobic metabolism such as lactate, which might be beneficial, it also diminished the vasoconstrictor responses to acute hypoxia, which might be deleterious for the fetus. This suggests that until further evidence is available, statins should be used with caution in pregnancy, particularly when poor placentation is evident.

In the UK, a double-blind, randomised placebo-controlled trial is currently studying the effect of pravastatin in women diagnosed with early-onset pre-eclampsia between 24 and 31 + 6 weeks of gestation (Table 1). The primary outcome measure is maternal sFlt-1 levels at 48 h after randomisation, with information also collected on maternal and neonatal mortality and morbidity. Another on-going double-blind, randomised placebo-controlled trial in the United States is investigating the safety and pharmacokinetics of pravastatin between 12 and 16 + 6 weeks of gestation in women with a history of pre-eclampsia (Table 1).^46^

### Plasma exchange and plasmaphaeresis

As many of the maternal manifestations of pre-eclampsia are produced by soluble mediators, removal of these factors is under investigation as a potential treatment. Plasma exchange, which is non-specific and non-selective, produced no benefit in early-onset pre-eclampsia (24–30 weeks of gestation, *n* = 7).^47^ Instead, it was associated with an initial worsening of hypertension, large volume ascites, pulmonary oedema, pleural effusions, and the need for delivery in all cases within 48 h of the intervention. Further studies using more selective approaches have shown potential benefit. Heparin-mediated extracorporeal LDL precipitation (HELP) removes a range of molecules including pro-atherogenic lipids, endothelin-1, tumour necrosis factor-alpha, homocysteine, and fibrinogen. In a pilot study in early-onset pre-eclampsia (24–32 weeks of gestation, *n* = 9) between one and seven sessions of HELP produced an improvement in blood pressure, proteinuria, and oedema in eight of the nine women, with an average interval to delivery of 17.7 days.^48^ More recently, dextran sulphate column aphaeresis was found to remove circulating sFlt-1 in women with pre-eclampsia.^49^ In three women who received repeated aphaeresis, the pregnancies were continued for 11 to 23 days. In some cases, however, aphaeresis reduced maternal blood pressure, with or without a fall in fetal heart rate. This resolved with maternal fluid administration, but it is unclear how growth-restricted fetuses might have responded, and further studies using longer term outcome endpoints are required.

## WHAT PRENATAL TREATMENTS CAN PREVENT THE ADVERSE OUTCOMES OF POOR PLACENTATION?

In the absence of a treatment, current obstetric practice offers some interventions to mitigate the adverse effects of poor placentation. Where iatrogenic preterm delivery is required, the use of prenatal corticosteroids to encourage fetal lung maturation and prevent respiratory distress syndrome^50^ and magnesium sulphate to reduce cerebral palsy, preserve gross motor function,^51^–^53^ and prevent eclampsia in at risk women is clearly of benefit.^54^–^56^ Interventions to promote smoking cessation should be offered where applicable,^57^ as smoking in the second and third trimester is associated with lower fetal weight, abruption, and poor neonatal outcome,^58^ which improves with smoking cessation.^59^–^61^ However, with the development of new prenatal interventions, it may become possible to further protect the fetus from the adverse intrauterine environment associated with poor placentation.

### Melatonin

Melatonin is a lipid soluble hormone released by the pineal gland, which has vasoactive, antioxidant, and immune-modulating properties. In newborns, it may have a neuroprotective effect following perinatal asphyxia,^62^ and melatonin administration during pregnancy is currently being investigated in the context of poor placentation, especially for its potential to reduce oxidative stress.

In a rat model of FGR, where maternal nutrient restriction from 15 dpc significantly reduces pup birthweight, maternal administration of melatonin from 15 dpc was associated with increased birthweight, comparable with that of control pups, and increased expression of the antioxidant enzymes magnesium superoxide dismutase and catalase.^63^ This effect was not replicated in a maternal nutrient restriction sheep model of FGR, where maternal melatonin treatment was associated with a non-significant reduction in fetal weight compared with control untreated nutrient-restricted dams and no difference in malondialdehyde (MDA), a marker of oxidative stress.^64^ However, maternal melatonin administration was associated with a significant increase in umbilical artery blood flow, both in adequately fed and nutrient-restricted dams, suggesting that it might have a beneficial effect on placental resistance.

A subsequent study used single umbilical artery ligation (SUAL) in sheep at 105 to 110 dpc (term = 145 dpc) to create asymmetric FGR, moderate fetal hypoxaemia, widespread white matter injury, and newborn developmental delay.^65^ Intravenous maternal melatonin administration starting 4 h after SUAL did not affect fetal growth but did significantly reduce markers of oxidative stress in the cerebral cortex and improve the time taken to achieve neonatal behavioural milestones, such as suckling.

A pilot study is currently on-going in Australia to investigate the effect of maternal melatonin administration on oxidative stress in pregnancies affected by FGR (Table 1).^66^ So far, six women have received melatonin with no adverse maternal or fetal effects, and this was associated with a significant reduction in placental MDA concentration compared with untreated women.^65^ A further Australian trial will be investigating whether maternal melatonin administration can delay the interval to delivery following a diagnosis of preterm pre-eclampsia (Table 1).^67^

### *N*-acetylcysteine

*N*-acetylcysteine (NAC) scavenges reactive oxygen species and forms the antioxidant glutathione, thereby counteracting oxidative stress and increasing the bioavailability of NO.^68^ In the rat RUPP model, NAC alleviated the rise in maternal blood pressure and increased pup brain weight when given from the time of arterial ligation.^69^ However, a small double-blind randomised controlled trial of NAC in early-onset severe pre-eclampsia or haemolysis, elevated liver enzymes, and low platelet count syndrome (HELLP) (25–33 weeks of gestation, *n* = 38) showed no difference in the interval to delivery, maternal complications, or neonatal outcome.^70^ This may be because it was too late in the disease course to be of benefit or because the dose of 600 mg TDS was not high enough. More recently, in a guinea pig model of maternal chronic hypoxia, administration of NAC did not affect pup weight but did ameliorate oxidative stress responses to hypoxia in the fetal liver.^71^ Further research is needed to investigate whether NAC may be useful in preventing fetal complications of poor placentation.

## HOW CAN NOVEL THERAPIES BE TRANSLATED INTO THE CLINIC?

To achieve real benefit for the future health of pregnant women, effective therapies must be brought from the realm of research into clinical practice. This process is hampered, however, by the reluctance of the pharmaceutical industry to invest in the development of new obstetric drugs or to licence existing drugs for use in pregnancy.^72^ This reluctance may arise from a fear of litigation combined with the practical and ethical complexity of conducting obstetric clinical trials, which must take into account not only the pregnant woman, the fetus, and long-term neonatal follow-up but also the diseases that currently have heterogeneous manifestations and unpredictable clinical progression. To some extent, safety considerations can be addressed with a firm basis of pre-clinical evidence. Ideally, this should include reproductive toxicology studies and evidence of efficacy in an animal model of the disease, taking into account species differences in placental structure and function as well as the timing of different stages of organ development.^73^,^74^ However, real progress will require greater recognition that excluding pregnant women from clinical trials does not protect them from risk but rather unfairly disadvantages them by restricting their access to evidence-based medicine.^75^–^77^

## CONCLUSION

Poor placentation causes significant maternal, fetal, and neonatal health problems in the short and long term. While clinicians currently have a handful of perinatal interventions to mitigate the adverse effects of poor placentation and associated iatrogenic preterm delivery, there are, as yet, no interventions that can increase fetal growth. An increasing understanding of the interlinked antiangiogenic mechanisms that underlie poor placentation, and its manifestation as pre-eclampsia and FGR, is allowing us to identify potential therapeutic targets. Translation into the clinic is complex and will require dialogue between patients, stakeholders, clinicians, and the regulators to ensure that the best possible outcomes are attained. The therapies that are currently being developed and tested offer hope for the future management of pregnancies affected by poor placentation, as well as providing a framework for the advancement of obstetric therapies as a whole.

### Ethics approval

As a review article, no research ethics application is required.

**WHAT'S ALREADY KNOWN ABOUT THIS TOPIC?**

- Poor placentation results in stillbirth, neonatal neurological, respiratory and gastrointestinal damage, and long-term health problems for affected women and surviving children.
- As yet, there is no treatment that can increase fetal growth *in utero*.

**WHAT DOES THIS STUDY ADD?**

- A number of therapies are showing potential to treat poor placentation, to improve fetal growth, and to prevent adverse neonatal outcomes. Clinical trials are planned or in progress to evaluate their safety and efficacy.
